# The Relationship Between Reading Strategy and Reading Comprehension: A Meta-Analysis

**DOI:** 10.3389/fpsyg.2021.635289

**Published:** 2021-08-04

**Authors:** Yuanke Sun, Jindao Wang, Yang Dong, Haoyuan Zheng, Jie Yang, Yaman Zhao, Weiyang Dong

**Affiliations:** ^1^Research Center for Overseas Studies and Media Reports on Hainan, Hainan Univeristy, Haikou, China; ^2^Department of Science and Environmental Studies (SES), The Education University of Hong Kong, Taipo, Hong Kong; ^3^Department of Teacher Education, Faculty of Education, Guangzhou Huashang College, Guangzhou, China; ^4^Department of English, College of Foreigh Language, Hainan University, Haikou, China; ^5^Department of Social and Behavioral Sciences, City University of Hong Kong, Kowloon, Hong Kong; ^6^Yew Chung International School - Primary HK, Kowloon, Hong Kong

**Keywords:** reading strategy, reading comprehension, discourse comprehension, elaboration strategy, organization strategy, monitoring strategy, affective strategy

## Abstract

This study synthesized the correlation between reading strategy and reading comprehension of four categories based on Weinstein and Mayer's reading strategy model. The current meta-analysis obtained 57 effect sizes that represented 21,548 readers, and all selected materials came from empirical studies published from 1998 to 2019. Results showed that reading strategies in all the four categories had a similar correlation effect size with reading comprehension. The correlation between monitoring strategy and reading comprehension was significantly larger in first language scripts than second language scripts. Affective strategy and elaboration strategy had an independent effect on reading comprehension, which was not significantly moderated by selected moderators. Results suggested that the reading strategies of all the four categories may have a similar contribution to text comprehension activities.

## Introduction

Reading comprehension requires readers to interpret the mental image from the given text through the interaction between both conceptual knowledge (e.g., vocabulary knowledge, metalinguistic knowledge) and procedural knowledge (e.g., reading strategy) and reading the text (Snow, 2002; Anmarkrud and Bråten, 2009; Daugaard et al., 2017). Regarding comprehension, reading strategies can be defined as a series of specific, deliberate, goal-directed mental processes or behaviors which control and modify the efforts of a reader to decode a text, understand words and construct the meaning of a text (Weinstein and Mayer's, 1986, p. 315; Anastasiou and Griva, 2009). Extensive literature suggests that reading strategies facilitate the text comprehension process through reading rate (Pattillo et al., 2004; McGeown et al., 2013; Lin and Yu, 2015), reading speed (Savaiano and Hatton, 2013; Alharbi, 2015; Layes et al., 2015), and comprehension accuracy (Aghaie and Zhang, 2012; Pei, 2014; Spörer and Schünemann, 2014). Weinstein and Mayer's (1986) categorized reading comprehension strategies into four groups based on strategy function: Affective strategy (AS), Elaboration strategy (ES), Monitoring strategy (MS), and Organization strategy (OS). Reading stages theory (Chall, 1983) provides the theoretical framework for reading purpose at different grade groups, from learning to read at lower grades of primary school, to becoming more professional in reading, to then be able to learn at the undergraduate level. However, the perspectives on the developmental relations (Kim et al., 2012; Quinn et al., 2015; Muijselaar et al., 2017), that is, the interaction effect between each reading strategy and reading comprehension on each reading stage remains unclear. Mayer (2005) cognitive theory of knowledge learning suggests that readers ought to employ more reading strategy levels in the first language (L1) than in the second language (L2). Readers should also employ more reading strategies in higher grades than in lower grades due to the fact that the background knowledge base is larger in higher grades than lower grades. However, previous literature shows inconsistent associations between each reading strategy and reading comprehension. Therefore, further exploration is needed on which category of reading strategy contributes more to reading comprehension. The extent of correlation between reading strategy and reading comprehension under the combined effect of reading stages and cognitive development remains unclear. Therefore, the current study employs a meta-analytic method to investigate the correlation between each reading strategy and reading comprehension. Furthermore, it investigates the effect of age effect and script differences effect on the correlation between each reading strategy and reading comprehension.

## Literature Review

### AS and Reading Comprehension

AS refers to a mental power that enables the reader to overcome negative feelings linked with the reading experience (e.g., reading anxiety). AS enhances the reading comprehension process through increasing performance goal attention time (Berthiaume et al., 2010), maintaining reading motivation level (Law, 2009; Logan et al., 2011; Schaffner and Schiefele, 2013), and controlling the negative experience effect (Guthrie et al., 2007; Bråten et al., 2013). For example, Lu and Liu (2015), Springer et al. (2017), and Wigfield et al. (2016) reported that the AS increased the reading interest and reading motivation of readers, and controlled feelings of boredom while reading. The assessment of AS included all possible strategies that decrease negative emotions and increase positive emotions or affective feelings (e.g., reading motivation strategy). However, previous studies showed various correlation levels between the AS and reading comprehension, from low (e.g., Bråten et al., 2013) to moderate (e.g., Law, 2009). Furthermore, it was unknown whether the various correlations could be explained by the reading stage or the level of cognitive development.

### ES and Reading Comprehension

ES refers to paraphrasing, summarizing, or describing the information of a mental image through existing knowledge on addressing target reading questions. ES establishes referential coherence, causal antecedents, and the emotion(al) reactions to the characters for both text-adjacent and global information inference, comprehension, and mental image construction (Cain et al., 2004; Currie and Cain, 2015; Daugaard et al., 2017). For example, ES establishes the local coherence between adjacent events and cues, constructing a global coherence among events and statements provided in the text (Long and Chong, 2001). The assessment of ES in a comprehension task requires the ability of the reader to integrate the target information among individual sentences in the text or the integration of general knowledge with information in the text (e.g., sentence-meaning inference). *Reading stages theory* suggests that students have a higher proficiency in ES due to the increase in background knowledge. However, previous studies showed that the association between the ES and reading comprehension varies from moderate (e.g., Cain et al., 2004) to high (e.g., Tsai et al., 2010). The developmental effect on the correlation between the ES and reading comprehension was also still unclear.

### MS and Reading Comprehension

MS refers to the ability to self-regulate or self-question the reading process and monitor the speed or quantity of the given text. MS is regarded as an essential metacognitive knowledge that supervises the reading state (e.g., the awareness on which categories of the content a reader needs to search) application (Baker and Brown, 1984; Kolić-Vehovec and Bajšanski, 2007). MS supervises the way that readers plan, evaluate, and utilize the available information from the text that makes sense of what they read from the literal textual information (Grabe and Mann, 1984; Dabarera et al., 2014). MS was assessed *via* supervision on the awareness of the reader on the reading progress. For example, MS was assessed by an error detection task, which required the detection of inconsistencies in the text that required readers to evaluate their understanding of the text and to regulate their reading to resolve any reading problems and to facilitate their understanding (Cain et al., 2004). Other assessments of comprehension monitoring included rating the awareness of the importance of sentences in a text, evaluating text complexity, and a cloze-task (Baker and Brown, 1984). Reading stages suggested that higher-level students should be more proficient with MS due to the increase in reading experience. However, previous studies reported that the association between the MS and reading comprehension varies from moderate (e.g., Taboada et al., 2009) to high (e.g., McNeil, 2011) at the same grade level. The effect of reading stages on the correlation between the MS and reading comprehension remains unclear.

### OS and Reading Comprehension

OS represents the ability to outline the passage or creating a hierarchy from the given reading text (Oakhill and Cain, 2012; Currie and Cain, 2015). OS is regarded as the determining factor in the explanation for comprehension failure or reading comprehension difficulties (Yap et al., 2012; Potocki et al., 2013). This means that the possible source of comprehension difficulties is inadequate knowledge about text structures. Past studies confirm explicit awareness about text structure, and the expectations engendered by certain common features of text may be useful aids for readers, helping them to invoke relevant background information and schemas to facilitate their construction of a meaning-based representation (Dabarera et al., 2014; Guajardo and Cartwright, 2016). The assessment of OS application includes passage organization identification and passage structure awareness (McNamara, 2004; Samuelstuen and Bråten, 2005). Higher proficiency in OS on reading comprehension needs more reading cognitive knowledge and reading experience (Guthrie et al., 2004; McNamara, 2004), suggesting the correlation between the OS and reading comprehension increases with reading experience and cognitive ability development. However, previous studies show variations in correlation between the OS and reading comprehension, from moderate (e.g., Samuelstuen and Bråten, 2005) to high (e.g., Guthrie et al., 2007). The effect between the reading stage and cognitive development on the correlation between the OS and reading comprehension requires further investigation.

### Potential Moderators

The current study selected grade group and language type as two potential moderators.

#### Grade Group

Reading stage theory (Chall, 1983) suggests that students start learning to read at early primary school and become more professional in reading to learn at university level. The different reading stages may result in students developing different levels of proficiency in reading strategies application. Past studies report that older readers performed better in ES on text comprehension, as readers were better able to explain the sorts of information that may be provided by the introduction and ending of a text (Adlof and Catts, 2015; Spencer and Wagner, 2018). Previous meta-analysis studies confirmed that the ability of reading procedural knowledge application was higher in higher-grade groups (e.g., Mol and Bus, 2011); the reason for this was identified as the larger interaction effect between literature knowledge and reading strategies, which was shown in higher-grade students than lower-grade students (Grabe and Mann, 1984; Berthiaume et al., 2010; Turnbull, 2016; MacSwan, 2017). Therefore, the grade group was selected as a potential moderator for this study.

#### Language Type

Mayer (2005) cognitive theory suggests that the proficiency in reading strategy application is higher in first language (L1) than second language (L2) reading comprehension, because readers obtain more background knowledge (e.g., vocabulary knowledge, grammatical knowledge, and word reading ability), which contributes text information coding and processing (Amadieu et al., 2009; Tarchi, 2015). Therefore, the current study selected language type as a potential moderator.

### The Current Study

To clarify the impact of the reading stage and the effects of cognitive developmental relations on the association between the four categories of reading strategy and reading comprehension, this study expanded the current literature concerning the impact of the four categories of reading strategy on reading comprehension through the meta-analytic method, exploring the correlation between four reading strategies and reading comprehension in different grade groups and language types. García and Cain (2014) showed that procedural knowledge of readers on comprehension develops faster before the master's degree learning period. Moreover, most students start formal reading comprehension at grade 1 of primary school (Law et al., 2008; García and Cain, 2014; Alharbi, 2015); therefore, the current study investigated grade group of students ranging from grade 1 of primary school to undergraduate level.

## Method

The current study followed official *PRISMA* guidelines on data collection and data analysis.

### Literature Base

This study selected dissertations, book chapters, and journal articles from a number of popular databases, such as CNKI, PsycINFO, ERIC, and EBSCO. Two groups of keywords were used for potential materials search. The first group of keywords related to strategy (reading strategy^*^, self-regulated reading^*^, memory reading^*^, monitor reading^*^, sentence verification^*^, word recognition^*^, inference^*^, predict^*^, sensitive^*^, letter knowledge^*^, reading skills^*^, mental skills^*^, meta-reading^*^, elaboration^*^, organization^*^, affective reading^*^, and psychological reading^*^). The second group of keywords related to reading comprehension (comprehension^*^, text comprehension^*^, passage comprehension^*^, paragraph comprehension^*^, sentence comprehension^*^, reading comprehension^*^, reading performance^*^, reading ability^*^, comprehension ability^*^, and reading acquisition^*^). All relevant materials published between January 1, 1998 and June 1, 2019, were selected, resulting in a total of 2206 articles.

### Inclusion Criteria

This study tried to include all possible studies that reported the correlation between reading strategy and reading comprehension. To control the possible cognition impact of the writing systems (García and Cain, 2014), this study only selected those that were written in Chinese or English. As a result, 183 articles were retained.

Regarding materials selection, both abstract and methodology were reviewed. Only the materials that met all the following requirements were entered into the database: (a) not case or review study; (b) materials should be empirical articles; (c) reading comprehension ability was reported by a specific measurement scores; (d) grade group of the participants ranged from grade 1 of primary school to undergraduate level; (e) participants without any diagnosed physical problem (e.g., deaf, blind); (f) materials provided the concurrent correlation indicator (correlation *r, R*^2^, *t*, and *p*-value) between reading strategy and reading comprehension, which could be transformed to Fisher's *z*, to investigate the concurrent correlation between reading strategy and reading comprehension; (g) the minimum number of participants was 30; and (h) provided a clear description on reading strategy, which can be coded by Weinstein and Mayer's (1986) *reading strategy theory*. Based on these criteria, this study removed 125 articles leaving 58 articles.

### Coding Process

Two independent coders coded the following information independently: (a) first author; (b) publication year; (c) sample size; (d) sampling area; (e) language type; (f) grade group; and (g) reading strategy category (AS, ES, MS, and OS). Regarding the reading strategy category, coders categorized reading strategy strictly according to the Weinstein and Mayer's (1986) reading strategy model definition. If the article only presented the general reading strategy score or the reading strategy category was not clear, the article was removed, because this study tried to investigate the correlation between each category of reading strategy and reading comprehension based on the reading strategy model. Any unclear information was emailed to the correspondence author of the article for confirmation. If the key information of the article (grade group, language type, and correlation indicators) was not clear, the article was removed. If one article provided more than two reading strategy categories (e.g., ES–reading comprehension, MS–reading comprehension), this study treated this article as two independent studies. If one article provided more than one available effect size in the same reading strategy category, this study ran the cluster regression method for effect size calculation (Hedges et al., 2010), thus ensuring that each study only provided one effect size (Mol and Bus, 2011). The intercoder reliability of coding was 0.96. The differences came from the sampling area coding. After discussion, this study solved the inconsistent results on sampling area coding through using the sampling country name to represent the sampling area. Two coders removed a total of 10 articles that were unclear in reading strategy category. All details of the remaining 48 articles are listed in Table 1.

**Table 1:** Description and outcomes in meta-analysis.

**No**.	**First author**	**Publication year**	***N***	**Language type^a^**	**Grade group^b^**	**Area**	**Strategy type^c^**	***z***	***SE***
1	Hannon	2012	150	1	U	USA	1	0.36	0.08
2	Zinar	2000	96	1	P	USA	3	0.48	0.10
3	Miller	2008	92	1	P	USA	1	0.50	0.11
4	Hannon	2001	69	1	U	Canada	1	0.43	0.12
5	Law	2009	120	1	P	HK	3	0.39	0.09
6	Hannon	2009	74	1	U	Canada	1	0.46	0.12
7a	Samuelstuen	2005	78	1	S	Norway	4	0.39	0.12
7b	Samuelstuen	2005	78	1	S	Norway	2	0.37	0.12
7c	Samuelstuen	2005	78	1	S	Norway	1	0.36	0.12
8	Jackson	2005	193	1	U	USA	1	0.45	0.07
9	Magliano	2011	190	1	U	USA	1	0.39	0.07
10a	Law	2008	837	1	P	HK	1	0.48	0.03
10b	Law	2008	837	1	P	HK	2	0.32	0.03
11a	Cain	2004	92	1	P	UK	1	0.44	0.10
11b	Cain	2004	102	1	P	UK	2	0.52	0.11
12	Leutner	2009	111	1	S	Germany	1	0.45	0.10
13	Koponen	2007	178	1	P	Finland	2	0.45	0.08
14	Onatsu	2000	105	1	P	Finland	3	0.42	0.10
15a	Taboada	2009	205	1	P	USA	2	0.40	0.07
15b	Taboada	2009	205	1	P	USA	3	0.42	0.07
16a	Tarchi	2015	166	1	S	Italy	2	0.39	0.08
16b	Tarchi	2015	166	1	S	Italy	1	0.37	0.08
17	Spörer	2009	186	1	S	German	1	0.37	0.07
18a	Dermitzaki	2008	127	1	P	Greece	2	0.62	0.09
18b	Dermitzaki	2008	127	1	P	Greece	3	0.59	0.09
19	McNamara	2006	39	1	S	USA	2	0.49	0.17
20a	Spörer	2009	210	1	P	German	1	0.44	0.07
20b	Spörer	2009	210	1	P	German	2	0.44	0.07
21a	Guthrie	2004	361	1	P	USA	4	0.76	0.05
21b	Guthrie	2004	361	1	P	USA	2	0.43	0.05
21c	Guthrie	2004	361	1	P	USA	3	0.44	0.05
22	Tompkins	2013	47	1	K	USA	1	0.32	0.15
23	Cain	1999	80	1	P	UK	1	0.46	0.11
24	Guthrie	2007	31	2	P	USA	3	0.45	0.19
25	McNeil	2011	20	2	U	USA	2	0.97	0.24
26	Tsai	2010	222	2	U	Taiwan	1	1.18	0.07
27	Prior	2014	53	2	S	Israeli	1	0.61	0.14
28	Logan	2011	111	1	P	UK	3	0.43	0.10
29	Kolić-Vehovec	2006	80	1	S	Croatia	1	0.29	0.11
30	Unsworth	2013	150	1	U	USA	3	0.42	0.08
31	Naseri	2012	59	2	U	Iran	2	0.51	0.13
32	Guthrie	1999	11738	1	S	USA	3	0.46	0.01
33	Braten	2013	65	1	S	Norway	3	0.42	0.13
34	Allen1	2014	108	2	U	USA	1	0.44	0.10
35	De Beni	2007	90	1	U	Italy	2	0.58	0.11
36	Tarchi	2010	149	1	S	Italy	1	0.45	0.08
37	Cromley	2010	737	1	U	USA	1	0.42	0.04
38	Hayashi	1999	100	2	U	Japan	3	0.20	0.10
39	Klauda	2008	278	2	P	USA	1	0.65	0.06
40	Wigfield	2008	492	2	P	USA	2	0.68	0.05
41	Motallebzadeh	2009	256	2	U	Iran	3	0.42	0.06
42	Zhang	2008	37	1	S	Singapore	1	0.46	0.17
43a	McNamara	2004	42	1	U	USA	1	0.44	0.16
43b	McNamara	2004	42	1	U	USA	4	0.54	0.16
43c	McNamara	2004	42	1	U	USA	2	0.45	0.16
44	Cromley	2007	175	2	S	USA	1	0.76	0.08
45a	Oakhill	2003	102	2	P	UK	1	0.44	0.10
45b	Oakhill	2003	102	2	P	UK	2	0.54	0.10
46	Zhang	2009	270	2	S	China	1	0.34	0.06
47	Hsu	2010	149	2	U	USA	3	0.34	0.08
48	Schaffner	2013	212	2	S	Germany	3	0.42	0.07

a *1, first language; 2, second language*.

b *K, kindergarten; P, primary school; S, secondary school; U, university*.

c *1, Elaboration; 2, Monitoring; 3, Affective; 4, Organizational*.

### Meta-Analytic Procedures

After inclusion and coding of materials, only 48 articles met all the requirements and were collected into the database for further analysis. All correlations between each category of reading strategy and reading comprehension scores were transformed into Fisher's *z* through inputting correlation indicator to Comprehensive Meta-Analysis. We selected Fisher's *z* for effect size because Fisher's *z* has asymmetrical distribution and the variance of Fisher's *z* is approximately constant (Borenstein et al., 2009). In general, the values of Fisher's z are 0.10, 0.31, and 0.55, which should be interpreted as that the effect size was small, moderate, and large, respectively (Cohen, 1988). If one of the selected articles measures the reading comprehension ability of students through both standardized and researcher-developed measurements, this study calculated the effect size from the standardized measurement. If one study provided more than one available measurement for the correlation indicator, this study ran cluster regression for effect size calculation (Hedges et al., 2010).

This study reported the effect size through the random-effects model from a conservative perspective, which provided the large range for correlation indicator estimation (Borenstein et al., 2009). Second, this study provided the 95% confidence interval (CI), and effect size should be interpreted as significant only if CI did not cross zero. Next, this study applied moderator analysis (e.g., meta-regression) when Chi-square value (*Q*-value) reached a significant level (*p* < 0.05). The meta-regression could only be used if the number of studies for analysis exceeded four (Hedges et al., 2010). Otherwise, this study applied sub-group analysis for the estimation of potential moderators (Borenstein et al., 2009).

Regarding publication bias examination, the current study used the funnel plot through the trim-fill method, rank correlation test, Egger's regression test, and Rosenthal's fail-safe number. Rosenthal's fail-safe number reflects the number of missing studies with null effects that would have to be retrieved and included in the analyses before the *p-*value becomes insignificant (Borenstein et al., 2009).

Durlak (2009) equation (*Teta*) was used for effect size comparison: Diff = Fisher's *z*_1_ - Fisher's *z*_2_, SE = Sqrt (Variance *z*_1_ + Variance *z*_2_). Teta = Diff / SE, if Teta ≥ 1.96 or Teta ≤ −1.96, the difference between Fisher' *z*_1_ and Fisher's *z*_2_ should be significant (*p* < 0.05).

## Results

### Descriptive Statistics

Four articles were removed due to the effect size over 3.5 standard deviation of the list (García and Cain, 2014). Specifically, the effect size of McNeil (2011) was removed from the MS–reading comprehension list and three effect sizes (Cromley and Azevedo, 2007; Klauda and Guthrie, 2008; Tsai et al., 2010) were removed from the ES–reading comprehension list. Finally, there were 44 articles with 57 effect sizes (*N* = 21,548). Specifically, 24 effect sizes (*N* = 4,163) related to the correlation between the ES and reading comprehension, 15 effect sizes (*N* = 3,078) related to the association between the MS and reading comprehension, 15 effect sizes (*N* = 13,826) related to the association between the AS and reading comprehension, and three effect sizes (*N* = 481) reported the correlation between the OS and reading comprehension. Twelve (12) effect sizes (*N* = 1,934) reported the correlation between L2 reading comprehension and reading strategy, and 45 effect sizes (*N* = 19,614) related to the correlation between L1 reading comprehension and reading strategy. Twenty-five (25) effect sizes (*N* = 5,591) reported the correlation between reading strategy and reading comprehension in primary school, 16 effect sizes (*N* = 13,506) related to the correlation between reading strategy and reading comprehension in secondary school students, and 16 effect sizes (*N* = 2,451) related to the correlation between reading strategy and reading comprehension in university students.

### Meta-Analysis 1

Table 2 provides each effect size between each category of reading strategy and reading comprehension. The effect sizes of reading strategies for all the four categories (AS–reading comprehension, ES–reading comprehension, MS–reading comprehension, and OS–reading comprehension) were close to large (Fisher's *z*_ES_ = 0.43, Fisher's *z*_MS_ = 0.48, Fisher's *z*_AS_ = 0.45, Fisher's *z*_OS_ = 0.58). The effect size comparison showed insignificant difference between every two categories of reading comprehension (*Teta* < 1.96, *p* >0.05). The Chi-square examination showed that the MS–reading comprehension and OS–reading comprehension correlations were significant (*Q*_MS−reading comprehension_ = 47.27, *p* < 0.001; *Q*_OS−reading comprehension_ = 9.27, *p* < 0.05). The moderator analysis showed that language type had a significant interaction effect on the correlation between the MS and reading comprehension (coefficient = 0.19, *p* < 0.01) with 77% variance explanation. Because only three studies were selected for the correlation between the OS and reading comprehension, sub-group analysis showed that the difference may be the grade group.

**Table 2:** Reading strategy and reading comprehension of four categories.

**Variables**	***k***	**Fisher's *z***	**95%CI**	***Q***	***N* fail-safe**	**Variance**	**Teta**
Affective	15	0.45	[0.44, 0.47]	12.45	3,622	<0.001	Teta _(Elaboration and Monitoring)_ = 1.44, Teta _(Elaboration and Affective)_ = 1.15, Teta _(Elaboration and Organization)_ = 1.14, Teta _(Monitoring and Affective)_ = 1.73, Teta _(Monitoring and Organization)_ = 0.74, Teta _(Affective and Organization)_ = 0.99
Elaboration	24	0.43	[0.40, 0.46]	11.91	3,693	<0.001	
Monitoring	15	0.48	[0.40, 0.55]	47.27^***^	2,118	0.001	
Organization	3	0.58	[0.32, 0.84]	9.27^*^	113	0.017	

* *p < 0.05*.

*** *p < 0.001*.

**Figure 1:**
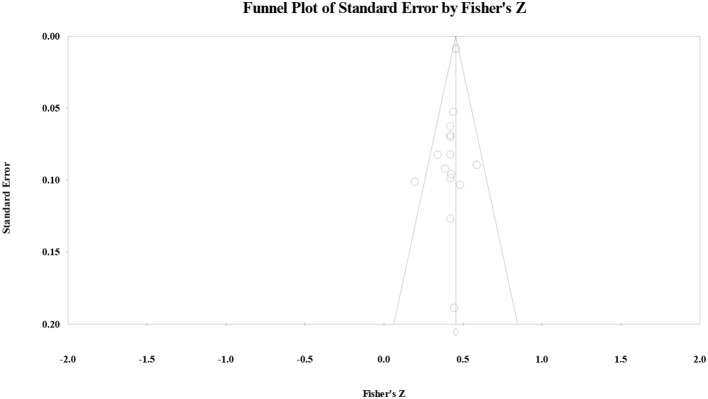
Funnel plot of the correlation between the affective strategy and reading comprehension.

Regarding publication bias examination, as for the correlation between the AS and reading comprehension, the funnel plot (Figure 1) showed that the effect size followed symmetric distribution, the safe-number was huge (*N* = 3,622), continuity Kendall's tau was.06 (*p* > 0.05), and Egger's regression intercept was −0.51 (*p* > 0.05). All four examination results suggested that the publication bias for the correlation between the AS and reading comprehension was not significant.

**Figure 2:**
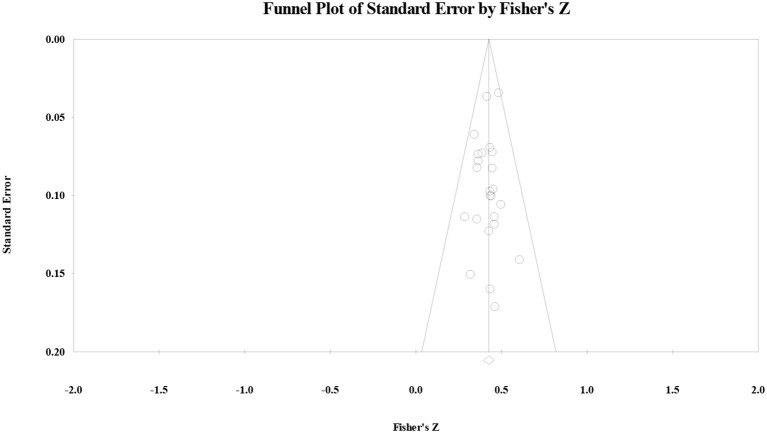
Funnel plot of the correlation between the elaboration strategy and reading comprehension.

As for the correlation between the ES and reading comprehension, the funnel plot (Figure 2) showed that the effect size followed a symmetric distribution, the safe-number was huge (*N* = 3,693), continuity Kendall's tau was 0.08 (*p* > 0.05), and Egger's regression intercept was −0.26 (*p* > 0.05). All four examination results suggested that the publication bias for the correlation between the ES and reading comprehension was not significant.

**Figure 3:**
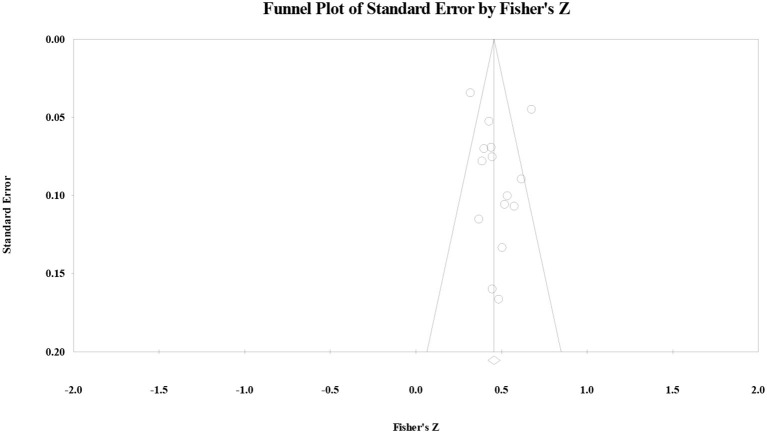
Funnel plot of the correlation between the monitoring strategy and reading comprehension.

As for the correlation between the MS and reading comprehension, the funnel plot (Figure 3) showed that the effect size followed symmetric distribution, the safe-number was huge (*N* = 2,118), continuity Kendall's tau was 0.15 (*p* > 0.05), and Egger's regression intercept was 0.93 (*p* > 0.05). All four examination results suggested that the publication bias for the correlation between the ES and reading comprehension was not significant.

**Figure 4:**
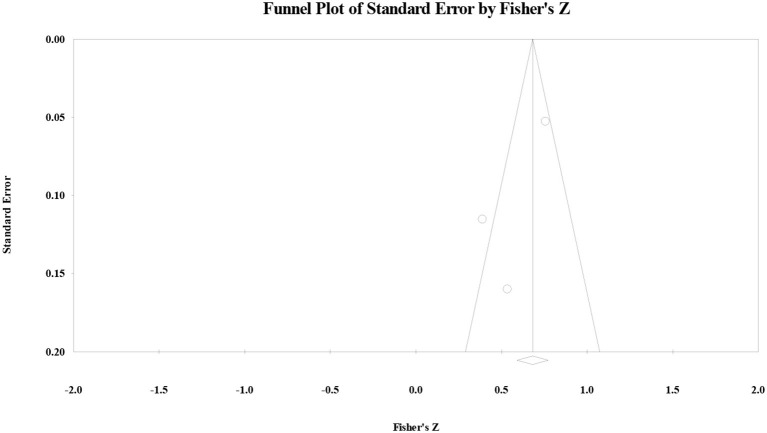
Funnel plot of the correlation between the organization strategy and reading comprehension.

As for the correlation between the OS and reading comprehension, the funnel plot (Figure 4) showed that the effect size followed symmetric distribution, the safe-number was huge (*N* = 113), continuity Kendall's tau was 0.33 (*p* > 0.05), and Egger's regression intercept was −3.42 (*p* > 0.05). All four examination results suggested that the publication bias for the correlation between the ES and reading comprehension was not significant.

### Meta-Analysis 2

The association between L1 monitoring strategy and reading comprehension was nearly large (Fisher's *z* = 0.43), and the Chi-square examination showed that heterogeneity within the 12 selected studies was not significant (*Q* = 16.59, *p* > 0.05). The association between L2 monitoring strategy and reading comprehension was large (Fisher's *z* = 0.63), the Chi-square examination showed that heterogeneity within the four selected studies was not significant (*Q* = 4.59, *p* > 0.05). The correlation between the MS and reading comprehension was larger in L1 than in L2 (Teta = 3.00, *p* < 0.001 see Table 3).

**Table 3:** Language type comparison on MS–reading comprehension.

**Variables**	***k***	**Fisher's *z***	**95% CI**	***Q***	**Variance**	***N* fail-safe**	**Teta**
L1	12	0.43	[0.38, 0.49]	16.59	0.001	1,136	Teta= 3.00
L2	4	0.63	[0.51, 76]	4.59	0.004	202	

## Discussion

The current study investigated the correlation between four reading strategies (AS, ES, MS, and OS) and reading comprehension. Results showed that each reading strategy had a similar effect size with reading comprehension. The AS–reading comprehension and ES–reading comprehension correlations did not moderate significantly by grade group and language type, suggesting that the reading stage and Mayer's cognitive theory had limited application on AS–reading comprehension and ES–reading comprehension. The effect size was larger in L2 than in L1 of the correlation between the MS and reading comprehension, extending Mayer's cognitive theory application on MS–reading comprehension: more background knowledge may inhibit the application on MS in reading comprehension. Sub-group analysis reported that the grade group may impact the correlation between the OS and reading comprehension, suggesting that the rule of the reading stage may influence the correlation between the OS and reading comprehension, higher-grade students should have more knowledge on text structure identification, but the association of OS–reading comprehension did not interact significantly by language type.

### Effect Size Comparison

Results showed that the four correlations between reading strategy (AS, ES, MS, and OS) and reading comprehension were similar, suggesting that different reading strategies may work together to contribute to the same comprehension task. For example, both OS and ES significantly predicted text main idea (e.g., McNamara, 2004; Samuelstuen and Bråten, 2005). Regarding behavior information process of text comprehension, this result informed us that there might be a reciprocal effect through all four categories' reading strategy on reading comprehension tasks, which means that students could improve their reading strategy knowledge in all the four categories by receiving any category of reading strategy training program. Results also informed that regardless of text categories (e.g., narrative, descriptive), readers needed to apply four reading strategies together for text comprehension in both surface and deep comprehension processes.

### AS and Reading Comprehension

The effect size between the AS and reading comprehension was nearly large and was not significantly impacted by selected moderators. This result is consistent with those reading affective/psychological factors studies (Lu and Liu, 2015; Rai et al., 2015; Arslan, 2017), which showed that processing text comprehension must experience negative emotion at the same time. Readers needed to overcome these negative emotions to finish the comprehension task. This result informed us that AS performed a vital role in negative emotion regulation on text comprehension, no matter at which reading stage or the level of cognitive load this took place. Results also informed that at any grades or language scripts text reading activities, OS were applied in these comprehension activities to enable the reader to overcome negative feelings linked with the reading experience.

### ES and Reading Comprehension

The current result was inconsistent with previous studies that showed the correlations between the ES and reading comprehension were different in different grade groups and L1 or L2 reading comprehension (e.g., Nation et al., 2010; Daugaard et al., 2017; Stanley et al., 2018). The correlation between the ES and reading comprehension was independent, which was not significantly moderated by the selected moderators. Reasons could be that the ES mainly contributed to the text mental information construction and integration process, which means that the ES has an independent working process whereby all different information needs to be abstracted from eyes first for coding, and then moved to the information integration process for comprehension. The current results showed that the grade group and language type did not have a significant impact on the correlation between the ES and reading comprehension through developmental relations, informing us that the ES might be the fundamental ability in text comprehension at any stage of reading and in any script of text comprehension. Results also informed that regardless of grades and language scripts comprehension activities, ES had similar contributions to printed text comprehension, enable readers to establish referential coherences, text semantic meaning identification, and mental image construction.

### MS and Reading Comprehension

The effect size in L2 was larger than in the L1 scripts. The familiarity of background knowledge on reading comprehension may be the main reason for the difference in the correlation between the MS and reading comprehension, because readers experienced more difficulties on L2 text comprehension due to less vocabulary knowledge, grammatical knowledge, and decoding ability than the L1 readers. Therefore, readers needed more cognitive resources on reading task supervision (Oakhill et al., 2003; Wigfield et al., 2008). This result reported that the supervision effect was different in different language scripts text comprehension, and readers needed to apply higher supervision effect on unfamiliar information processes.

### OS and Reading Comprehension

The effect size of the correlation between the OS and reading comprehension was large, revealing that the text structure awareness played a key role in main idea identification (e.g., Samuelstuen and Bråten, 2005), which echoed findings of previous studies on the impact of awareness on text comprehension process. Moreover, moderator analysis showed that the grade group might explain the main reason for various correlations between the OS and reading comprehension across different reading stages. Previous studies pointed out that the OS would be applied more frequently in a higher-grade group than in younger groups for more complexity of the comprehension task (Cain et al., 2004; Guthrie et al., 2004; Silva and Cain, 2015). In the higher grade group, the requirement of the comprehension task is higher and the structure of the task comprehension gains more complexity, so higher proficiency in OS application contributed to completing the comprehension task. These results inform us that at the reading-to-learn stage, readers gain more professional awareness of text structure on reading comprehension tasks.

### Limitations

The current study has several limitations. First, it did not involve students who were diagnosed with serious special education needs (e.g., blind, deaf). Second, this study only synthesized those studies written in Chinese or English, and those studies written in other languages were not involved. Past studies have already demonstrated that the different language scripts cognition effect (e.g.,transparent vs. opaque) would enhance or inhabit text comprehension process (Wydell, 2012; Filippello et al., 2016; Rappo et al., 2017). Finally, this study reported the effect size between each reading strategy and reading comprehension based on Weinstein and Mayer's (1986) reading strategy model. However, the number of empirical studies on organization strategy effect was limited.

## Conclusions

This study derived conclusions from the combined results of 57 effect sizes that represented more than 20,000 students. To summarize, this meta-analysis has confirmed that AS, ES, MS, and OS contributed similar effect size on the text comprehension process, implicating all the four reading strategies had closed interactions and collaborations which contributed to text comprehension activities together. The reading stage statement might impose limitations on AS and ES on text comprehension application. Readers had greater awareness of L2 reading progress supervision than L1. Correlation between the OS and reading comprehension might be impacted by reading stage, and higher-grade readers performed with higher OS proficiency on reading comprehension task.

## Author Contributions

YS draft the manuscript. JW provided the dataset and instructions on draft writing. YD contributed to data analysis and report writing. HZ provided ideas in a theoretical framework construction. JY, YZ, and WD provided comments and suggestions on manuscript revision. All authors contributed to the article and approved the submitted version.

## Conflict of Interest

The authors declare that the research was conducted in the absence of any commercial or financial relationships that could be construed as a potential conflict of interest.

## Publisher's Note

All claims expressed in this article are solely those of the authors and do not necessarily represent those of their affiliated organizations, or those of the publisher, the editors and the reviewers. Any product that may be evaluated in this article, or claim that may be made by its manufacturer, is not guaranteed or endorsed by the publisher.
